# “He was no longer listening to me”: A qualitative study in six Sub-Saharan African countries exploring next-of-kin perspectives on caring following the death of a relative from AIDS

**DOI:** 10.1080/09540121.2018.1537467

**Published:** 2018-10-25

**Authors:** Robert Ssekubugu, Jenny Renju, Basia Zaba, Janet Seeley, Dominic Bukenya, William Ddaaki, Mosa Moshabela, Joyce Wamoyi, Estelle McLean, Kenneth Ondenge, Morten Skovdal, Alison Wringe

**Affiliations:** a Rakai Health Sciences Program, Kampala, Uganda; b Population Studies Group, Faculty of Epidemiology and Public Health, London School of Hygiene and Tropical Medicine, London, UK; c Malawi Epidemiology and Intervention Research Unit, Karonga, Malawi; d Faculty of Public Health and Policy, London School of Hygiene and Tropical Medicine, London, UK; e MRC/UVRI and LSHTM Uganda Research Unit, Entebbe, Uganda; f Africa Health Research Institute,South Africa; g University of KwaZulu-Natal, Durban, South Africa; h National Institute for Medical Research, Mwanza Centre, Mwanza, Tanzania; i Kenya Medical Research Institute Center for Global Health Research Kisumu, Kenya; j Department of Public Health, University of Copenhagen, Copenhagen, Denmark; k Biomedical Research and Training Institute, Harare, Zimbabwe

**Keywords:** HIV, caregiving, care-receiving, qualitative, PLHIV, sub-Saharan Africa

## Abstract

In the era of widespread antiretroviral therapy, few studies have explored the perspectives of the relatives involved in caring for people living with HIV (PLHIV) during periods of ill-health leading up to their demise. In this analysis, we explore the process of care for PLHIV as their death approached, from their relatives’ perspective. We apply Tronto’s care ethics framework that distinguishes between care-receiving among PLHIV on the one hand, and caring about, caring for and care-giving by their relatives on the other. We draw on 44 in-depth interviews conducted with caregivers following the death of their relatives, in seven rural settings in Eastern and Southern Africa. Relatives suggested that prior to the onset of poor health, few of the deceased had disclosed their HIV status and fewer still were relying on anyone for help. This lack of disclosure meant that some caregivers spoke of enduring a long period of worry, and feelings of helplessness as they were unable to translate their concern and “caring about” into “caring for”. This transition often occurred when the deceased became in need of physical, emotional or financial care. The responsibility was often culturally prescribed, rarely questioned and usually fell to women. The move to “care-giving” was characterised by physical acts of providing care for their relative, which lasted until death. Tronto’s conceptualisation of caring relationships highlights how the burden of caring often intensifies as family members’ caring evolves from “caring about”, to “caring for”, and eventually to “giving care” to their relatives. This progression can lead to caregivers experiencing frustration, provoking tensions with their relatives and highlighting the need for interventions to support family members caring for PLHIV. Interventions should also encourage PLHIV to disclose their HIV status and seek early access to HIV care and treatment services.

## Introduction

Although antiretroviral therapy (ART) provision across sub-Saharan Africa has reduced adult mortality (Reniers et al., 2017), premature deaths among people living with HIV (PLHIV) continue to occur (Slaymaker et al., 2017). In many settings, the burden of caring for PLHIV prior to their demise falls to relatives. The family’s role in caring for a member living with HIV can be an invaluable social capital resource (Homan, Searle, Esu-Williams, Aguirre, & Mafata, 2005) but also a safety net with holes (Seeley et al., 1993). Family members often play a crucial role in providing care at home for relatives, reminding them to go to clinic (Skovdal, Campbell, Madanhire, Nyamukapa, & Gregson, 2011), take their medication (Beals, Wight, Aneshensel, Murphy, & Miller-Martinez, 2006) and providing palliative care when close to death (Skovdal, Ogutu, Aoro, & Campbell, 2009). However, the relationship between caregivers and receivers is complex. Carer obligations are sometimes driven by fear of guilt in the event their relative dies (Zhou, 2010), while individuals receiving family support can feel obliged to maintain their health, thereby lightening the burden for their helpers (Ware et al., 2009).

Deaths among PLHIV in the ART era suggest insurmountable barriers to care or ART adherence, whether related to health care provision or support structures, including the family (Croome, Ahluwalia, Hughes, & Abas, 2017). However, while there is evidence of health systems and community issues influencing HIV care engagement, the role family members have been relatively under-explored. Furthermore, most qualitative studies investigating HIV care engagement focus on the perspectives of PLHIV and health workers, with few considering relatives’ experiences. Research that does consider relatives’ perspectives, draws on an ethics of care framework to make visible the caring practices and interactions that are mobilised within family structures. These studies have identified HIV care interdependencies in African settings (Evans & Atim, 2015; Skovdal et al., 2018), with some noting how conflicting emotional connections between care providers and receivers may constrain “good care” (Evans & Thomas, 2009), and shape the availability of resources to provide and receive care, particularly in contexts of stigma, and social and economic inequality. We contribute to the expansion of the ethics of care framework in HIV care, exploring the perspectives of next-of-kin who have lost a relative to AIDS.

Tronto (1993), in theorising ethics of care, identifies four ethical elements, each characterised by certain moral qualities. In the first phase, “caring about”, the relative may recognise unmet needs for care and exhibit a level of *attentiveness* to these needs. In the second phase, “caring for”, or “taking care of”, the relative begins to additionally take *responsibility* for the care of the PLHIV, often assuming the burden of care. In the third phase, “care-giving”, the relative not only shoulders the responsibility of care, but also begins the actual work of providing “good care”, requiring necessary skills and competence. During each phase, the relative observes how the PLHIV responds to their care, i.e., “care-receiving”. Being able to react, adjust and advance their care, based on these reactions and responses requires the responsiveness of the relative for caregiving to continue. This framework allows an exploration of the influences on the evolution of the care relationships that existed between the caregivers and their recently deceased relatives, and has proved to be particularly useful in disentangling the morality and dynamics of care in the contexts of HIV and stigma (Evans & Atim, 2015; Evans & Thomas, 2009). More recently, Tronto (2013) has proposed a fifth phase of care, namely “caring with”, referring to the politics of availing care support in ways that uphold democratic commitments to justice, equality and freedom for all. The inclusion of the fifth phase allows us to explore the gendered nature of care and critically examine the need for interventions to support family members of PLHIV.

## Methods

### Study locations and participants

We draw on data from the “Bottlenecks Study” that examined how PLHIV interacted with HIV services in seven health and demographic surveillance sites (HDSS) in sub-Saharan African settings, namely Karonga (Malawi), Rakai and Kyamulibwa (Uganda), Kisesa (Tanzania), Kisumu (Kenya), Manicaland (Zimbabwe), and uMkhanyakude (South Africa) (Wringe, Renju, Seeley, Moshabela, & Skovdal, 2017).

### Sampling and recruitment

Each HDSS regularly conducts regular verbal autopsy (VA) interviews with household members using structured questionnaires to ascertain likely cause of death for deceased persons in the surveillance area (Floyd et al., 2012). VA interviews were conducted by trained health workers who identify symptoms and events leading up to a death. The VA tool includes an unstructured narrative component to document additional information, including whether, to the respondent’s knowledge, the deceased had been diagnosed with HIV or had attended an HIV clinic. Each site provided a sampling frame including study identifiers and contact information for participants who had completed the VA tool for a deceased relative and (approximate) date of their death, restricted to participants who reported awareness of the deceased’s HIV-positive status. Sampling ensured that the deceased had a range of HIV treatment histories.

### Data collection

Between October 2015 and April 2016, we conducted face-to-face in-depth interviews in the local language with relatives of PLHIV who had died from HIV-related causes within the past twelve months to four years (Table 1). Topic guides drew on the VA narrative and covered the relationship of the caregiver with the deceased, their interactions, and their perspective on their relative’s life and illness including experiences of health service use (if any). Interviews lasted between 45 and 90 min and took place in a private location selected by the caregiver.

**Table 1.** Characteristics of the care-givers.

Characteristics	*n* (%)
Sex	
Male	14 (32%)
Female	30 (68%)
Age	
Average male	49 years old
Average female	50 years old
Overall average	50 years old
Age range	20–79 years old
Occupation	
Formal employment	3 (9%)
Informal activities	41 (91%)
Relation to deceased	
Father	2 (5%)
Mother	3 (7%)
Cousin	1 (2%)
Son	8 (18%)
Daughter	3 (7%)
In-law	3 (7%)
Brother	8 (18%)
Sister	5 (11%)
Husband	2 (5%)
Wife	4 (9%)
Other relatives	5 (11%)
Sampling site	
Rakai (Uganda)	8 (18%)
Kyamulibwa (Uganda)	5 (11%)
Kisumu (Kenya)	11(25%)
Kisesa (Tanzania)	5 (11%)
Karonga (Malawi)	6 (14%)
��Manicaland (Zimbabwe)	3 (7%)
uMkhanyakude (South Africa)	6 (14%)

Interviews were audio-recorded following written, informed consent, and either transcribed and translated (uMkhanyakude, Manicaland, Kisumu, Rakai, Kisesa, Karonga) or summarised into detailed field reports (Kyamulibwa). Data were anonymized and securely stored in password-protected files. Pseudonyms are used in this paper.

### Data analysis

A latent thematic analysis was conducted whereby patterns in the data were identified inductively and considered systematically. A broad analytical framework was collaboratively developed based on the emerging themes which broadly aligned with Tronto’s ethics of care framework (Tronto, 1993). All the transcripts were coded in Atlas.ti version 5.2.0 (Berlin, Germany). Emerging hypotheses were regularly discussed with each site’s study coordinator and team.

### Ethical considerations

Ethical approval was granted in each country and by the London School of Hygiene and Tropical Medicine.

## Results

A total of 44 caregivers were interviewed. In each HDSS, except Karonga, the majority were female and were 20–67 years (Table 1). Caregivers were predominantly small-scale farmers. The deceased were predominantly male (61%), and most were reported to have initiated ART (Table 2).

**Table 2:** . Characteristics of the deceased.

Characteristics	*n* (%)
Sex	
Male	27 (61%)
Female	17 (39%)
Age	
<40	12 (27%)
40+	12 (27%)
Unknown	9 (20%)
Location when care-giver first recalled relative was sick.	
Same/nearby village	19 (43%)
Far from home	18 (41%)
Different country	3 (7%)
Unspecified	13 (30%)
ART status	
Had never initiated ART	2 (9%)
Had initiated ART	30 (66%)
Unclear as to whether had initiated ART	1 (0.2%)

### Being concerned and “caring about”

Prior to the onset of very poor health, few of the deceased had disclosed their HIV status and fewer still relied on anyone for help. Some respondents felt that they were deliberately kept out of the loop, and only their relative’s changing behaviour or daily practices raised their suspicions:

He did not disclose that he had tested positive. It was when his wife had given birth to this child that she said she had been told that she will breastfeed the child for only six months. I was shocked and asked her why  … . … and she said that was what she had been advised. They did not want to disclose. (71 years, mother to the deceased, southern Africa)

This lack of disclosure, or running away, meant that some caregivers endured long periods of worry and feelings of helplessness, unable to translate their concern and “caring about” into “caring for”. For some caregivers, their concern about their relative’s health were rooted in observations of deteriorating health, for others it was the sudden “running away” or refusal to receive care or discuss their health that sparked their suspicion, cemented by concerns raised by friends or partners of the deceased.

Josiah moved and spent a long time away. We were worried if he was going to come back and whether he was alive […] we were called by his friends telling us that they worried about my brother’s health condition. Josiah would fall down and become unconscious and after some time he would regain consciousness. (36 years, brother to the deceased, southern Africa)

### Taking responsibility and “caring for”

The transition from “caring about” to also “caring for” was often characterised by tangible actions, such as visiting relatives, bringing the PLHIV back home, or following up on treatment. Caregivers’ accounts frequently illustrated a sense of duty and responsibility to care for their relative. The responsibility was often culturally prescribed, rarely questioned and usually fell to women. One woman described the burden of care for her husband following his diagnosis:

I encouraged Mrisho to take an HIV test, I took him to hospital, … Mrisho was just okay but he was hungry and then he also had a headache, and he had a high fever and I undressed him, cooked for him and he ate, and he went to sleep, I gave him ‘panadol’ (painkillers) and he slept. (26 years, wife of the deceased, eastern Africa)

The transition from “caring about” to “caring for” was often accompanied by some acceptance, on the part of the sick family member of their need for care. This absence of notable resistance enabled caregivers to take responsibility and “care for” their relatives, corresponding with initial experiences of care receiving on the part of the sick family member. Nevertheless, many respondents recounted regular struggles to mobilise resources to take on caring responsibilities, which could lead to resentment. A few months after Faraja had interacted with her son, David, she received a call from the lakeshore where he fished, and learnt that he was sick, leaving her to weigh up competing demands on the family’s limited resources:

I started getting calls every time that I needed to go and collect David and that they [his acquaintances] didn’t want him to die at the lake shore. During that time, one of my sons (Baraka) was planning to get married and we had already spent a lot of money to buy the items that were demanded from the bride’s side. We were only left with one cow which we were planning to slaughter for that function. Really that cow was not ours, it was for Baraka who was planning his marriage, but we decided to sell it so that we could get money for transport to Kalangala [where David was] and back. (44 years, mother of the deceased, eastern Africa)

### Being able to offer “care-giving”

The transition from “caring for” to “care-giving” was characterised by recognising and having the necessary skills and competences to carry out care which could include more intensive, physical acts of providing care for their relative. Family members were often the only people available and capable of offering care on a daily basis, including ensuring that their medical care needs were met.

In addition to supervising pill-taking, care-giving also involved either cooking, or spoon-feeding the sick relative, taking them to hospitals, helping them bathe and use the toilet, and sometimes physically restraining them where they were described as mentally ill or drug-dependent. One sister of a deceased person spoke of her strategies to overcome the resource limitations that she faced, as well her competence and skills in offering effective care.

I encouraged and took Julius for HIV testing, I took him to hospital when he got very sick and stayed with him at the hospital, I looked around for funds to buy the required drugs. So, I stayed there to take care of Julius. They could tell me to buy drugs and I did not have enough money. Some of the family members could send me some money. I would use this to buy drugs and then smash them up with some food and give him, but he could hardly take them. Julius could only eat something very little. (32 years old, sister of the deceased, eastern African region)

During this stage of care, care-receiving was often characterised by conflict and tensions between caregiver and unwell family member. Caregivers often referred to the deceased as giving up, losing hope, and worrying (expressed as “thinking too much”). Caregivers were often faced with resistance, enduring verbal abuse, and had to adapt to mood swings:

He kept telling me ‘what have you seen in me that makes you tell me to go to the hospital?’ So it was never easy.  … he was no longer listening to me. (66 years old, mother of the deceased, eastern Africa)

Such tensions arose if a sick family member was perceived to be renouncing their responsibility for maintaining their good health by deliberate non-adherence to their medication. Tensions were further perpetuated if their sick family members’ behaviour caused an additional burden on financial resources, or a need for physical or palliative care. In many instances, this led to the caregiver adopting a dominant role where they were often perceived by their relatives as being “pushy” or bothering them.

Caregivers used other creative ways to counteract the resistance to care that they faced from their relatives, including going directly to healthcare providers and requesting them to test their relative for HIV or to follow them up. In other cases, they turned to third parties to persuade their relative to take their medication. Whilst this was sometimes apparently effective, it could compromise confidentiality and erode trust between caregivers and their relatives, perpetuating existing tensions in the caregiver-care receiver relationship.

 … Takondwa did not agree to take medication and I even brought some women who were on ART to talk to her. There is a certain woman who came to Takondwa and told her “rise up, do you want to die here? Don’t you see we are on HIV treatment and we are doing well and even looking after our children? Don’t lie to people that you are sick yet you know what you are suffering from. Why can’t you seek HIV care and treatment?. (43 years, mother to the deceased, eastern Africa)

## Discussion

Our study adds to research exploring the experience of providing palliative care for PLHIV in rural African settings, confirming the physical, emotional and financial burden that intense caring can entail (Mburu et al., 2013). The heavy emotional burden felt by many of our participants, particularly women, was not only linked to the loss of a relative, but also due to the inherent tensions in the caregiving and care-receiving relationships between many PLHIV and family members prior to their deaths. These tensions were often rooted in the lack of acceptance of HIV status, non-disclosure, and shame that characterise these particularly vulnerable PLHIV, and led to their resistance to care-receiving.

**Figure 1.**
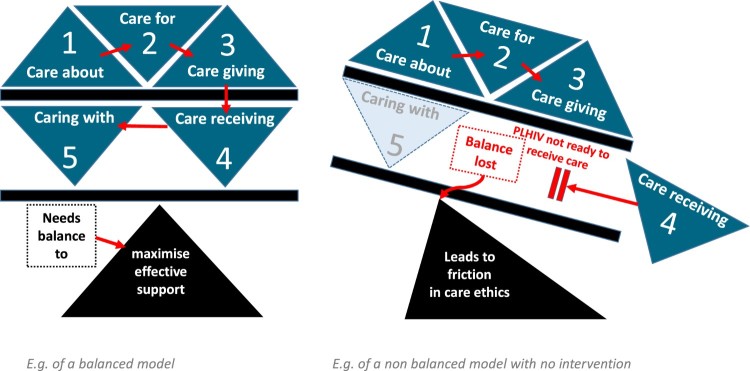
Ethics of caregiving model as it relates to support of and acceptance of support by PLHIV A balanced model of care b. an example of a non-balanced model

By applying Tronto’s framework, we distinguished between different phases of caring that occur during the illness of a household member before their demise. This conceptualisation of the caring relationship highlights how the burden of caring often grows more intense as family members caring evolves from “caring about”, to “caring for”, and eventually to “giving care” to their relatives. During the latter stages of ill-health, the potential reciprocities in caring were most likely to fall out of balance, such that caregiving became challenging or impossible, and care-receiving by PLHIV became insufficient and ineffective (Figure 1).

Whilst other studies on caring for PLHIV have shown that caregivers may experience pride and enrichment through their actions and diligence to their cultural responsibilities (Williams, 2002), in our study settings, it was more often frustration, anger and disillusionment that were expressed by participants, sometimes leading to tense relationships with their relatives. One explanation is that many studies on caring experiences were conducted prior to the ART roll-out, when little could be done to prevent these untimely deaths. In the ART era, caregivers’ frustrations when faced with relatives who refuse to disclose their status, or to take, or to adhere to treatment, may override any sense of moral satisfaction in providing end-of-life care. The role of a caregiver is usually unexpected, and generally the caregivers are neither socialised nor prepared (Pereira & Rebelo Botelho, 2011).

Our findings build on other analyses that have unpacked the underlying tensions that can occur between family members and PLHIV who reject their attempts to give them care, often fuelled by underlying poverty and the pervasive stigma associated with HIV (Bonnington et al., 2017). Skovdal et al. (2017) have noted how men living with HIV may be seen as “rebellious” and “irresponsible” by relatives by either refusing care that is on offer, or by refusing to enter into the reciprocal arrangement of care-giving and care-receiving as proposed by the caregiver. Other studies have also highlighted how notions of responsibility that are projected onto PLHIV to care for themselves by getting diagnosed, and engaging with the demands of HIV treatment may carry moralistic undertones of blame, further stigmatising these individuals and further undermining any attempts to enable them to engage with HIV care (Bond et al., 2016). Our findings suggest interventions should acknowledge the gendered nature of “care with” family members, enabling them to live full and healthy lives, while simultaneously allowing them to look after sick PLHIV (Tronto, 2013). Such interventions must address the financial burden of care, while recognising the psychological and physical aspects of caring for relatives who resist medical interventions (Herce et al., 2014).

Various study limitations should be considered, including the fact that we draw on the perspectives of caregivers who had completed the VA tool, who are often the head of household, but not necessarily the person who gave most care to their relative who died. Furthermore, interviews were conducted once, meaning that we were unable to explore any evolution in participants’ views. Finally, we did not access the HIV status of caregivers, and this characteristic could influence the way in which they experience and describe care-giving.

## Conclusions

In conclusion, through family members’ accounts of recently deceased PLHIV, our study provides unique insights into caring dynamics in households prior to a HIV-related death. These caring relationships are often fraught by increasing tensions that arise due to the financial, emotional and physical burden of caring for a relative who is often an unwilling recipient of care. Interventions are needed to support family members caring for PLHIV at the end of their lives, as well as for PLHIV who struggle to overcome persistent social and structural barriers to disclosure and timely access to available HIV care services.

We would like to thank all the participants and fieldworkers who contributed their time and effort to the study. We would also like to acknowledge the support of ALPHA representatives at each HDSS who facilitated the implementation of the fieldwork and many other colleagues within the ALPHA Network who made helpful suggestions throughout the design and conduct of the research.
